# A cross-sectional controlled developmental study of neuropsychological functions in patients with glutaric aciduria type I

**DOI:** 10.1186/s13023-015-0379-6

**Published:** 2015-12-22

**Authors:** Nikolas Boy, Jana Heringer, Gisela Haege, Esther M. Glahn, Georg F. Hoffmann, Sven F. Garbade, Stefan Kölker, Peter Burgard

**Affiliations:** Department of General Paediatrics, Division of Child Neurology and Inherited Metabolic Diseases, Centre for Paediatrics and Adolescent Medicine, University Hospital Heidelberg, Im Neuenheimer Feld 430, D-69120 Heidelberg, Germany; Faculty of Applied Psychology, SRH University of Applied Sciences, D-69123 Heidelberg, Germany

**Keywords:** Glutaric aciduria type I, Cognitive development, Information processing, Dystonia

## Abstract

**Background:**

Glutaric aciduria type I (GA-I) is an inherited metabolic disease due to deficiency of glutaryl-CoA dehydrogenase (GCDH). Cognitive functions are generally thought to be spared, but have not yet been studied in detail.

**Methods:**

Thirty patients detected by newborn screening (*n* = 13), high-risk screening (*n* = 3) or targeted metabolic testing (*n* = 14) were studied for simple reaction time (SRT), continuous performance (CP), visual working memory (VWM), visual-motor coordination (Tracking) and visual search (VS). Dystonia (*n* = 13 patients) was categorized using the Barry-Albright-Dystonia Scale (BADS). Patients were compared with 196 healthy controls. Developmental functions of cognitive performances were analysed using a negative exponential function model.

**Results:**

BADS scores correlated with speed tests but not with tests measuring stability or higher cognitive functions without time constraints. Developmental functions of GA-I patients significantly differed from controls for SRT and VS but not for VWM and showed obvious trends for CP and Tracking. Dystonic patients were slower in SRT and CP but reached their asymptote of performance similar to asymptomatic patients and controls in all tests. Asymptomatic patients did not differ from controls, except showing significantly better results in Tracking and a trend for slower reactions in visual search. Data across all age groups of patients and controls fitted well to a model of negative exponential development.

**Conclusions:**

Dystonic patients predominantly showed motor speed impairment, whereas performance improved with higher cognitive load. Patients without motor symptoms did not differ from controls. Developmental functions of cognitive performances were similar in patients and controls. Performance in tests with higher cognitive demand might be preserved in GA-I, even in patients with striatal degeneration.

**Electronic supplementary material:**

The online version of this article (doi:10.1186/s13023-015-0379-6) contains supplementary material, which is available to authorized users.

## Background

Glutaric aciduria type I (GA-I) is caused by autosomal recessive mutations in the *GCDH* gene (cytogenetic location: 19p13.2) resulting in glutaryl-CoA dehydrogenase (GCDH; EC 1.3.99.7) deficiency) [1] and elevated concentrations of glutaryl-CoA, glutaric acid (GA), 3-hydroxyglutaric acid (3-OH-GA) and glutarylcarnitine (C5DC) in body tissues. GA and 3-OH-GA are considered to be neurotoxic [2–5]. Two biochemical subtypes, excreting low vs. high concentrations of GA and 3-OH-GA in urine have been delineated [6]. Cerebral accumulation of GA and 3-OH-GA is induced by cerebral *de novo* synthesis and low efflux transport of these metabolites across the blood–brain barrier [7–9].

Delayed brain maturation starting in the last trimester of pregnancy results in temporal hypoplasia, immature gyral patterns and delayed myelination [10–13]. The prognostic relevant clinical presentation is a predominantly dystonic movement disorder due to striatal injury which may manifest insidiously or acutely after a preceding acute encephalopathic crisis [11, 14, 15], usually within the first three years of life. Early diagnosis and start of metabolic treatment comprising low lysine diet, carnitine supplementation and emergency treatment during episodes that are likely to induce catabolism like febrile illness can prevent striatal injury and improve neurological outcome [15–20].

Whereas susceptibility to striatal injury is restricted to a vulnerable period in infancy and early childhood, extrastriatal MRI changes such as T2 hyperintensity of supratentorial white matter may increase with age [11, 21]. These changes correspond to spongiform myelinopathy due to myelin splitting and intramyelinic vacuolation as shown by post mortem studies [22–24]. Severe white matter changes are the characteristic finding in GA-I patients with a late-onset disease variant. However, white matter changes may already start during childhood – even in patients identified by newborn screening [11, 25, 26].

Neuropsychological function comprises several steps of information processing from encoding of stimuli, working memory, decision making, attention and organization of motor function. These steps require structural and functional integrity of specific gray and white matter, and circuits including cortex, basal ganglia and thalamus, i.e. cortico-striato-thalamocortical loop [27]. Given the characteristic cerebral abnormalities in GA-I and in analogy to white matter changes in other neurological diseases [28], patients might be at risk for neurobehavioral impairment of reaction times, information processing or abnormal development of cognitive functions. Basal ganglia injury, frontotemporal cortical hypoplasia and accumulating neurotoxins might further increase this risk [11, 27, 29–31].

Most studies on GA-I were performed in paediatric patients and focussed on motor dysfunction. The general assumption is that intellect is spared in GA-I [32]. In fact, normal cognitive function was reported, however, studies had only small sample sizes and no control group [33]. However, decreased IQ or more subtle cognitive deficits were also reported for a few children with GA-I [34, 35] and cognitive decline was found in late-onset GA-I patients [26], The few studies on cognitive function in GA-I only focussed on IQ but not on development of cognitive function. Given the ubiquitous results that even well-treated patients with GA-I show frequent neuroradiological abnormalities [11, 36] the present study investigates a broader spectrum of neuropsychological functions from childhood to adulthood.

The aims of the present study were (1) to analyse the development of neuropsychological functions in symptomatic as well as asymptomatic GA-I patients focussing on information processing measuring simple reaction time, continuous performance, visual working memory, visual motor coordination and visual search using a computer-based test battery and to compare the results with age-matched healthy control group data, and (2) assessment of the impact of dystonic movement disorder on test results.

## Methods

### Study samples

We investigated 30 patients (12 female and 18 male) with confirmed diagnosis of GA-I who are followed by our own department or are members of the German GA-I patient group (http://www.glutarazidurie.de). Only patients of at least 5 years of age were included in the study (age range 5–29 years) due to our clinical experience that younger children usually are not able to perform all subtests of our battery or to cope with a total test time of 60 minutes. Patients with dystonia in their hands unable to manage the response panel of our test battery did not participate. Patients’ data were compared with those of healthy subjects (*n* = 196; age range 5–28 years, 93 female and 103 male). Control group data for children and adolescents have been collected in local schools and for adults at workplaces requiring different educational levels before this study for evaluation of other metabolic diseases, e.g. phenylketonuria. Detailed information on age group sizes of patients and controls are given in Additional file 1: Table S1.

The study was approved by the Institutional Ethics Committee of the Medical Faculty of Heidelberg University (No. 314/2002; S-49/2010; S-525/2010). Data were collected after written informed consent had been obtained from GA-I patients or their parents as well as control subjects and were pseudonymized before statistical analysis.

### Outcome parameters

#### Dystonia

Severity of dystonia was determined using the Barry-Albright Dystonia Scale (BADS, [37]). The BADS is a five-point ordinal scale (0 = no dystonia; 1 = slight; 2 = mild; 3 = moderate; 4 = severe) quantifying dystonia in eight body regions (eyes, mouth, neck, trunk, right and left upper and lower extremities), by a score ranging from 0 to 32. Accordingly, subjects without motor symptoms received a BADS score of zero.

#### Reaction times and information processing

The computer-based neuropsychological test battery used for this study was compiled by two of the authors (PB, SG) for testing patients with inherited metabolic diseases [38, 39]. The aim of the battery is to test basic processes of information processing mostly devoid of educational influences using the same tasks across the age range from childhood to adulthood. The five subtests measure different basic aspects of information processing using standard neuropsychological paradigms [40, 41]. The test battery comprises pure motor speed tests (simple visual reaction time, visual motor coordination), speed tests with low (continuous performance) or increasing (visual search 1,2,3) cognitive demand, and a test without time constraints and high cognitive demand (visual working memory). The tracking and the continuous performance tests measure level of performance as well as stability of performance over time. Five tests were administered individually to each patient.

In the *simple reaction time* (SRT) task a sequence of 80 black squares appears on the screen and requires pushing a button as fast as possible. Inter-stimulus intervals are randomized to prevent strategy effects. Test scores are calculated as the mean of 80 reactions (40 with the dominant and 40 with the non-dominant hand). *Visual*-*motor coordination* (Tracking) was measured with a smiley moving on the computer screen which has to be pursued with the mouse cursor as close as possible. To increase difficulty mouse cursor movements are attenuated, i.e. slightly delayed in time. Test scores are mean distance between the cursor and the smiley for speed and SD of the distance for stability of performance. *Continuous performance* (CP) was measured in a choice reaction time paradigm. A sequence of 600 patterns of three, four or five squares is presented. A button for the dominant hand has to be pushed after four squares (hits) and a button for the non-dominant hand after three or five squares. Test scores are mean reaction time for hits measuring level of performance and standard deviation of single hit times measuring stability of performance. In *Visual search* (VS) the time to decide whether or not a squared pattern of four different letters contained a set of target letters was measured. Tested are three loads of one (VS1), two (VS2) or three target letters (VS3). Test scores were mean reaction times for correct choices in the three loads. In the *visual working memory* (VWM) task a target checker board pattern of 64 black or white squares is presented for one second. After an inter-stimulus interval the target is presented together with three distractors (same size checker boards with different patterns). The subject has to decide which of the options shows the target stimulus, and to choose it by a mouse click. There is no time limit for decision making. The first ten trials are used to adaptively define a subject’s baseline performance of discrimination, where the target-distractor similarity is stepwise increased or decreased according to the subject’s performance. In the following ten trials the subject’s test performance is determined by the mean number of different fields for correct differentiations between target and distractors (mean contrast).

### Statistical analysis

Raw scores for all psychological tests were converted to standard deviation scores (SDS) based on means and standard deviations of age-matched healthy control groups, higher SDS indicating better performance. The age range (5–29 years) was discretized into five age groups of two years each for ages 5 to 14 years, and five age groups of three years each for ages 15 to 29 years. Data of all psychological tests were log-transformed because of non-normal distributions of raw scores.

**Fig 1:**
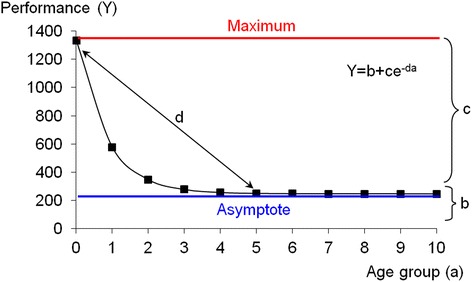
A general negative exponential model of development. The model [42, 43] describes development of performance (Y) as an exponential function of age (a). b represents the asymptote to which the performance converges, c is the difference between maximum of Y and asymptote. d indicates how quickly performance changes from maximum to asymptote

Parameters of a mathematical model of developmental [42, 43] (Fig. 1) were calculated for the cross-sectional results of GA-I patients and controls separately. The model describes developmental change by a negative exponential function of performance Y by age (a) as Y = b + ce^-da^. Parameter *b* represents the asymptote to which performance converges over time, *c* is the difference between the maximum of Y and the asymptote and *d* indicates how fast Y changes from maximum to the asymptote. For curve fitting of the model to the data nonlinear regression was used [44, 45]. Akaike weights were used as criterion for global optimisation of the model parameters b, c, and d. The Akaike Information Criterion corrected for bias (AICc) is recommended to measure goodness of fit in non-linear modelling [46]. Unfortunately, AICc is not standardized like R^2^, but can only be interpreted as relative measure, with low values indicating good and high values indicating poor fit. We therefore also report residual sums-of-squares (RSS) indicating better vs. poorer goodness of fit. As AICc depends on sample size, values for the control group are in a different numerical range than those of the patient group and subgroups.

Since the three cognitive loads of the VS task (VS1-VS3) cannot be simultaneously handled in a single developmental model, data have been analysed as follows. Repeated ANOVA of VS1-VS3 by group and age group revealed significant effects for group [F(1,187) = 13.24, *p* < 0.001], with patients being slower than controls, age group [F(2,187) = 62.36, *p* < 0.001], with older subject being faster (a common developmental trend), and load [F(1.6,303.3) = 183.27, *p* < 0.001, Greenhouse-Geiser], with more letters (higher cognitive demand) leading to longer response times (a normal effect). The interaction of load by age group was significant [F(3.2,303.3) = 7.14, *p* < 0.001, Greenhouse-Geiser]. Compared with younger subjects older ones needed less additional time from VS1 to VS2 to VS3, what can be expected, and is compatible with a shift from serial to parallel information processing.

The interactions of load by group [F(1.6,303.3) = 0.55, *p* = 0.543, Greenhouse-Geiser] and load by group by age group [F(3.2,303.3) = 0.20, *p* = 0.908, Greenhouse-Geiser] were not significant. Thus, increase of reaction time from VS1 to VS3 and decrease of reaction time with age are comparable in both groups. The non-significant interaction of load by group indicates that differences between patients and controls are the same across all three loads. Therefore, each load can be regarded as representing the group effect. We selected VS3 for deriving a developmental function for visual search.

Mathematical models resulting for patients and controls were compared by the F-Test described by Mead and Curnow [47]. Due to the explorative nature of the analysis no adjustment of alpha-error was performed. *P* values ≤ 0.05 were considered statistically significant, values > 0.05 and ≤ 0.1 are reported as trends.

## Results

### Study sample

Fourteen of the 30 GA-I patients were diagnosed by newborn screening (median age at diagnosis 0.25 months, range 7–17 days), 12 patients were diagnosed by targeted metabolic studies after manifestation of a movement disorder (median age at diagnosis 37 months, range 2–111 months), and three patients were diagnosed by high-risk screening of families with previously diagnosed index patients (one patient at the age of 1 month, one patient at the age of 53 months; the exact date of diagnosis of one patient being unknown). An additional patient was diagnosed coincidentally at the age of 25 years participating in a diagnostic study. Eight patients, four asymptomatic (24 %) and four symptomatic (31 %) did not complete all five subtests. This difference was not significant [χ^2^(df = 1, *n* = 30) = 0.20, *p* = 0.485]. Numbers and reasons of unfinished tests for each subtest were for VS: *n* = 3 asymptomatic patients: lack of concentration *n* = 1, young child without familiarity with letters *n* = 1, unknown *n* = 1; *n* = 4 symptomatic patients: young child without familiarity with letters *n* = 1, fatigue with increasing dystonia: *n* = 1, unknown: *n* = 2; for VWM: *n* = 1 asymptomatic patient: lack of concentration; *n* = 2 symptomatic patients, both unknown; for CP: *n* = 1 asymptomatic patient: fatigue after MRI and sedation the day before; *n* = 1 symptomatic patient: fatigue with increasing dystonia. SRT and Tracking subtests were finished by all patients.

Thirteen patients (43 %) had a dystonic movement disorder, eight of them classified as mild (BADS score = 1-7), two as moderate (BADS score = 8-14) and three as severe dystonia (BADS score > 14). In all dystonic patients, both upper extremities were involved. Seventeen patients (57 %) were asymptomatic (BADS score = 0).

### Effects of dystonia on test results

To elucidate impact of dystonia on motor reaction times, we correlated BADS scores with neuropsychological test results. BADS scores were unavailable for three patients with dystonia due to missing documentation. Since BADS scores for upper extremities significantly correlated with total BADS scores (*r* = 0.918; *p* < 0.001, *n* = 27), only the latter were used for further statistical analysis. We found a monotonically decreasing function of performance in psychological tests starting with BADS scores equal three. Correlations were significant between BADS scores and speed tests with low cognitive and high motor demand (means of SRT and CP, mean and SD of Tracking; Table 1). The speed test with high cognitive demand (VS1 VS2, VS3), the test with high cognitive demand without time constraints (VWM) and the stability of the test with low cognitive demand (SD of CP) were not correlated with BADS scores. The difference between the results of the two stability variables for Tracking and CP will be elaborated in the discussion. Partial correlations controlling for age did not change the pattern of associations.

**Table 1:** Pearson correlation coefficients between neuropsychological tests and BADS scores

Test	Construct	Variable	Correlation		
			Pearson r	p	n
Tracking	Speed (motor demand)	SDS Mean	−0.720	<0.001	27
Tracking	Stability (motor demand)	SDS SD	−0.722	<0.001	27
SRT	Speed (motor demand)	SDS Mean	−0.505	0.007	27
CP	Speed (low cognitive demand)	SDS Mean	−0.467	0.021	24
CP	Stability (low cognitive demand)	SDS SD	−0.096	0.654	24
VS 1	Speed (high cognitive demand)	SDS Mean L1	−0.255	0.264	21
VS 2	Speed (high cognitive demand)	SDS Mean L2	−0.346	0.135	20
VS 3	Speed (high cognitive demand)	SDS Mean L3	0.059	0.811	19
VWM	High cognitive demand (without time constraint)	SDS Mean Contrast	−0.062	0.775	24

### Developmental functions for psychological data

Table 2 reports residual sums-of-squares (RSS) indicating better vs. poorer goodness of fit. As AICc depends on sample size, values for the control group are in a different numerical range than those of the patient group and subgroups. There were good fits of the exponential model to the data for all psychological test scores, both for patients and controls, except for VWM where AICc and RSS values were largest for patients and controls. This was in accordance with our expectation as the developmental model is not designed for variables without time constraints [42, 43].

**Table 2:** Parameter estimates of the exponential model with F-test for comparison of groups

Test	Variable	Model parameter		Parameter estimate (PE)	SE of PE	t-test for PE	p value of t-test	RSS	AICc		PE	SE of PE	t-test for PE	*P* value of t-test	RSS	AICc	F (df1, df2)	*p*	F (df1, df2)	*p*
			n	GA-I patients						n	Controls						Pat vs. Controls		Asymp. vs. Controls	
SRT	Log Mean	b	30	2.38	0.07	33.95	<0.001	0.362	−38	196	2.38	0.01	309.48	<0.001	1.014	−467	3.40 (3,220)	0.019	−0.63 (3,207)	0.595
		c	30	0.58	0.11	5.33	<0.001			196	0.92	0.13	7.05	<0.001						
		d	30	−0.46	0.24	−1.8	0.071			196	−0.92	0.11	−8.15	<0.001						
CP	Log Mean	b	27	0.88	0.05	16.81	<0.001	0.508	−22	183	0.75	0.03	25.37	<0.001	1.231	−388	2.57 (3,204)	0.056	0.54 (3,193)	0.657
		c	27	1.01	0.44	2.32	0.030			183	0.62	0.04	16.41	<0.001						
		d	27	−0.91	0.45	−2.00	0.057			183	−0.31	0.05	−5.91	<0.001						
Track-ing	Log Mean	b	30	1.40	0.05	29.61	<0.001	0.444	−32	119	1.38	0.02	81.71	<0.001	1.458	−178	2.63 (3,143)	0.052	3.64 (3,130)	0.015
		c	30	0.62	0.30	2.07	0.049			119	0.87	0.15	5.88	<0.001						
		d	30	−0.80	0.52	−1.53	0.137			119	−0.72	0.14	−5.26	<0.001						
VS 3	Log Mean L3	b	21	3.01	0.07	46.22	<0.001	0.596	−6	172	2.88	0.03	107.14	<0.001	2.362	−241	2.93 (3,187)	0.035	2.32 (3,179)	0.077
		c	21	1.63	0.69	2.37	0.029			172	0.97	0.12	8.20	<0.001						
		d	21	−0.90	0.39	−2.31	0.033			172	−0.46	0.08	−5.92	<0.001						
VWM	Log Mean Contrast	b	27	0.69	0.17	4.10	<0.001	1.603	9	125	0.66	0.03	24.80	<0.001	5.923	−18	1.90 (3,146)	0.133	0.61 (3,135)	0.609
		c	27	0.73	0.25	2.89	0.008			125	1.62	0.64	2.53	0.013						
		d	27	−0.43	0.42	−1.03	0.314			125	−1.13	0.35	−3.27	0.001						
				Dystonic (Dys.) patients							Asymptomatic (Asymp.) patients						Dys. vs. Asymp.		Dys. vs. Controls	
SRT	Log Mean	b	13	2.43	0.06	39.30	<0.001	0.131	−12	17	2.36	0.08	30.36	<0.001	0.218	−16	0.30 (3,24)	0.824	4.67 (3,203)	0.004
		c	13	0.90	0.38	2.39	<0.038			17	0.80	0.27	3.01	0.009						
		d	13	−0.75	0.43	−1.75	0.112			17	−0.65	0.39	−1.65	0.122						
CP	Log Mean	b	11	0.85	0.05	17.21	<0.001	0.076	−12	16	0.84	0.15	5.61	<0.001	0.382	−4	0.75 (3,21)	0.533	4.85 (3,188)	0.003
		c	11	1.39	0.50	2.76	0.025			16	0.59	0.22	2.68	0.019						
		d	11	−0.95	0.37	−2.59	0.032			16	−0.46	0.50	−0.92	0.377						
Track-ing	Log Mean	b	13	1.41	0.06	21.81	<0.001	0.158	−10	17	1.29	0.09	15.01	<0.001	0.231	−15	1.14 (3,24)	0.351	0.08 (3,126)	0.969
		c	13	1.00	0.47	2.15	0.057			17	0.70	0.24	2.92	0.011						
		d	13	−0.80	0.47	−1.69	0.123			17	−0.60	0.42	−1.43	0.175						
VS 3	Log Mean L3	b	8	2.50	10.02	0.25	0.813	0.192	7	13	3.00	0.09	31.97	<0.001	0.356	1	n.d.	n.d.	n.d.	n.d.
		c	8	0.78	9.59	0.08	0.939			13	1.72	0.82	2.10	0.062						
		d	8	−0.06	0.97	−0.06	0.956			13	−0.90	0.47	−1.91	0.085						
VWM	Mean Contrast	b	11	0.64	0.34	1.88	0.100	0.652	12	16	0.79	0.10	8.10	<0.001	0.845	8	0.49 (3,21)	0.690	2.09 (3,130)	0.105
		c	11	0.91	0.50	1.80	0.110			16	6.35	47.34	0.13	0.895						
		d	11	−0.36	0.53	−0.69	0.513			16	−2.81	7.57	−0.37	0.717						

F-test according to Mead and Cournow 1983. Data of all psychological test scores show good fits to the model (R^2^_adj_ ≥ 0.442), both for GA-I patients (*n* = 30; asymptomatic: *n* = 17; dystonic: *n* = 13) and controls (*n* = 196), with the exception of VS3 for dystonic patients. For definition of model parameters b,c and d see Fig. 1. R^2^_adj_: adjusted explanatory power of the regression model. SE: standard error

**Fig. 2:**
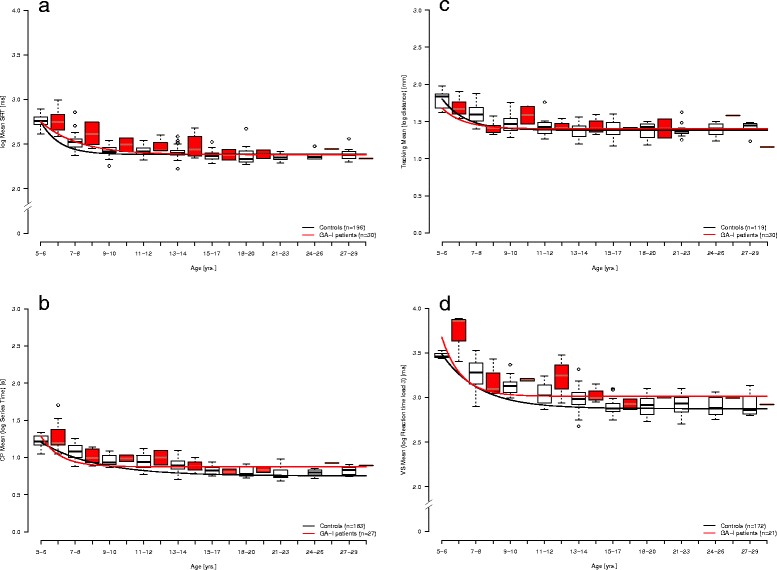
**a–d**. Developmental functions for SRT (**a**), CP (**b**), Tracking (**c**) and VS3 (**d**) of GA-I patients and controls. Patients (red) and controls showed significant differences for SRT and VS3 and an obvious trend for CP and Tracking (for numeric details see Table 2). Height of box: interquartile range; bottom end of box: Q1; top end of box: Q3; bold line inside of box: median; bottom/top whiskers: lowest/highest case within 1.5 times interquartile range; circles = outliers: distance to Q1 or Q3 respectively ≥ 1.5 and ≤ 3 times interquartile range

**Fig. 3:**
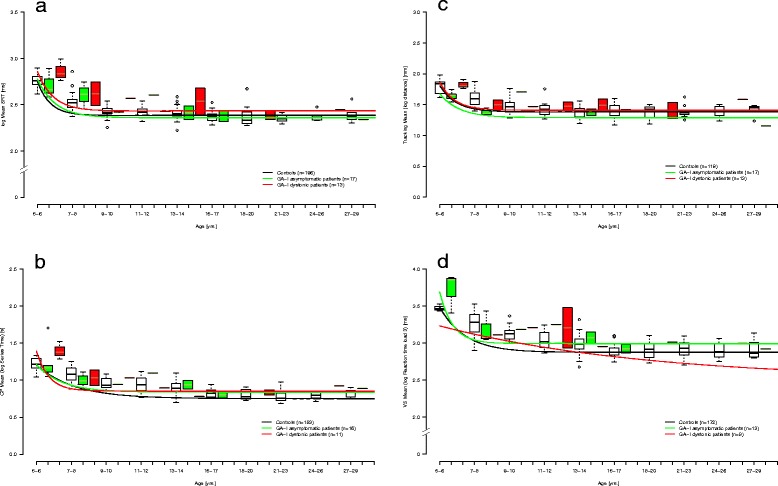
**a–d**. Developmental functions for SRT (**a**), CP (**b**), Tracking (**c**) and VS3 (**d**) of asymptomatic and dystonic GA-I patients and controls. Dystonic patients (red) were slower in SRT and CP. Asymptomatic patients (green) did not differ from controls (black), except significantly better results in Tracking and a trend for slower reactions in VS3. Height of box: interquartile range; bottom end of box: Q1; top end of box: Q3; bold line inside of box: median; bottom/top whiskers: lowest/highest case within 1.5 times interquartile range; circles = outliers: distance to Q1 or Q3 respectively ≥ 1.5 and ≤ 3 times interquartile range. For numeric details see Table 2

Comparison of developmental functions Y = b + ce^-da^ of patients vs. controls (Fig. 2a-d) using the F-test [47] yielded significant differences for SRT (*p* = 0.019) and VS3 (*p* = 0.035), and trends for CP (*p* = 0.056) and Tracking (*p* = 0.052). No significant difference was found for VWM (*p* = 0.133) (Table 2). Furthermore, we compared developmental functions for asymptomatic and dystonic patients with those of controls and against each other (Fig. 3a-d). In SRT, dystonic patients showed longer reaction times than controls (*p* = 0.004). Differences between dystonic and asymptomatic patients (*p* = 0.824) as well as between asymptomatic patients and controls were not significant (*p* = 0.595) (Table 2). For CP, a similar pattern was found with only dystonic patients being significantly slower than controls (p = 0.003) whereas asymptomatic patients did not differ from controls. In the Tracking task, dystonic patients were not significantly different from asymptomatic ones (*p* = 0.351) or controls (p = 0.969), and asymptomatic patients showed significantly faster reaction times than controls (*p* = 0.015). VS3 which was selected for deriving a developmental function for visual search resulted in a good fit for patients as well as for healthy control subjects. We observed a trend for asymptomatic patients showing slower reaction times than controls (*p* = 0.077). The dystonic subgroup was too small to achieve an acceptable fit for the exponential model (Fig. 3d). Developmental functions for VWM, a pure cognitive demand test without time constraints, showed highest AICc and RSS values and poor fits for GA-I patients and controls. No developmental functions were derived for the two stability variables, as no specific hypothesis was formulated for change of variability with age.

To test whether the exponential functions of the three groups show different gradients (Fig. 1, parameter d, i.e. how fast the asymptotic performance is achieved), patient and control data were aggregated into three different age groups: a starting period (5–8 years) with a relatively low level of performance, followed by a period of rapid improvement (9–12 years), and a period of relatively stable performance without further improvement (13–29 years). A significant interaction of group by age would indicate different gradients. No significant interactions could be found for SRT, CP, Tracking, VS3, or VWM (Table 3).

**Table 3:** ANOVA test variables by group (controls, asymptomatic patients, dystonic patients) and age group (5–8, 9–12, 13–29 years)

Test	Variable	Source	Test result
SRT	Log Mean	Group	F(2,217) = 8.07, *p* < 0.001
		Age group	F(2,217) = 58.48, *p* < 0.001
		Interaction (group by age group)	F(4,217) = 1.49, *p* = 0.208
CP	Log Mean	Group	F(2,201) = 1.63, *p* = 0.198
		Age group	F(2,201) = 52.48, *p* < 0.001
		Interaction (group by age group)	F(4,201) = 1.04, *p* = 0.390
Tracking	Log Mean	Group	F(2,140) = 0.71, *p* = 0.496
		Age group	F(2,140) = 22.65, *p* < 0.001
		Interaction (group by age group)	F(4,140) = 1.44, *p* = 0.223
VS3	Log Mean L3	Group	F(2,184) = 3.62, *p* = 0.029
		Age group	F(2,184) = 15.99, *p* < 0.001
		Interaction (group by age group)	F(4,184) = 1.54, *p* = 0.192
VWM	Log Mean Contrast	Group	F(2,143) = 2.60, *p* = 0.078
		Age group	F(2,143) = 13.02, *p* < 0.001
		Interaction (group by age group)	F(4,143) = 0.61, *p* = 0.658

## Discussion

This is the first study analysing different aspects of information processing in a larger group of GA-I patients. We have found five major results: (1) Dystonia correlated significantly with motor speed. (2) In contrast, performances with higher cognitive load (visual working memory and visual search) as well as stability of performance over time were not associated with dystonia. (3) Developmental functions of GA-I patients were different from those of healthy controls for motor speed and visual search but not for visual working memory (a test with higher cognitive demand without speed constraints). Dystonic patients showed significantly longer reaction times compared to controls in simple motor speed and continuous performance but were not different in visual motor coordination and visual search. (4) Overall, developmental functions of speed tests of all three groups fitted well with the negative exponential model. Differences between groups were mainly due to level but not to gradient, i.e. dystonic patients were slower from the start. The interaction of group by age group (Table 3) was non-significant for all tests, indicating that dystonic patients reached their asymptote of performance as fast as asymptomatic patients and controls, i.e. although the three groups do not develop on the same level, the form of their development is the same (in analogy to growth development on different percentiles). (5) Performance of asymptomatic patients did not significantly differ from controls, except for visual motor coordination, where asymptomatic patients showed significantly better results, and visual search, where asymptomatic patients showed a trend for slower reaction times.

### Effects of dystonia on test results

The differential association between BADS scores and information processing is a strong argument for the internal validity of our test battery. Speed tests are susceptible for motor impairment whereas tests with higher cognitive demand without time constraints are not. The difference in the results of the two stability measures can be explained by specific facets of the tasks. Stability in *Tracking* is measured by the distance of the mouse pointer from the moving smiley, i.e. high stability is achieved by continuous adaptation of the direction of the mouse pointer to the randomly changing direction of the smiley. In contrast, stability in continuous performance (*CP*) is measured by variation of reaction times in a repetitive motor task. Overall slower and faster subjects can have the same stability, e.g. by remaining on their relative speed level during the whole test. Dystonic patients also showed longer reaction times in motor speed (SRT) and CP due to their motor handicap. Notably, in visual working memory (VWM), a test without time constraints, dystonic patients did not differ from asymptomatic patients and controls. These results show (1) that it is possible and necessary to separate speed (motor) and non-speed information processing, and (2) that cognitive functions might be preserved even in symptomatic GA-I patients.

### Development of neuropsychological functioning in GA-I patients

In analogy to growth development described by anthropometric parameters on growth charts, age related changes of reaction time, information processing and other cognitive functions can be described as developmental functions. Mean differences of level of performance may be clinically important, however, impaired development like developmental arrest or loss of function would result in different developmental trajectories.

The model of negative exponential developmental function [42, 43] fitted well with the data for both patient groups and healthy controls. Across all domains investigated, asymptomatic patients showed normal development. Dystonic patients also showed a normal gradient over time and age, however, on a slower level for speed tests.

For all patients, VWM was not different from controls. SRT (slower in dystonic patients) and VWM (similar results in patients and controls) are on the extremes of the speed vs. cognitive demand dimension of our tests and the pattern of our results, speed being affected in dystonic patients, and VWM performance being normal in dystonic as well as asymptomatic patients, supports the hypothesis that intellect might be preserved in GA-I [32], and neurological disease manifestation may be limited to dystonic movement disorder or subtle neurological abnormalities. In line with our findings Brown et al. [33] recently showed that IQ scores and executive functioning were normal in six GA-I patients diagnosed by newborn screening. Our concept of working memory does not include the cognitive manipulation of the stored stimulus. However, it requires the serial comparison of a stored image with presented stimuli by segmenting the target and the distractors into similar and dissimilar parts, going beyond a pure short-term memory task. The requirement of designs using more complex cognitive paradigms is discussed in the limitations paragraph.

Recent observations also revealed subtle or global intellectual impairment in GA-I patients identified by newborn screening [34–36], cognitive decline in adults with late-onset GA-I [26] and white matter changes in MRI progressing with age [11] as a result of spongiform myelinopathy [22–24]. Memory deficits have not been reported for GA-I patients – except for a 66-year-old patient with late-onset disease manifestation presenting with a progressive neurologic disease including focal seizures, hand tremor, ataxia, and progressive dementia [26] and another patient identified by newborn screening who was born prematurely and owing to significant social issues was taken into foster care [34]. In line with this, we found similar patterns for VWM in patients and controls. Interestingly, deficits in long- and short-term memory were recently demonstrated in Gcdh-deficient mice [48].

#### Normal development in spite of harmful cerebral conditions

In GA-I, numerous cerebral abnormalities have been described, inborn and acquired, structural and functional, that may potentially disturb cognitive function. Structural cerebral abnormalities comprise temporal hypoplasia and white matter changes which might already start in early childhood [11, 25, 26]. Temporal hypoplasia, cortical immaturity and delayed myelination might already start in the last trimester of pregnancy [10–13]. Temporal hypoplasia is found in the majority of asymptomatic and dystonic patients [11] but relevance for cognitive performance has not been studied in detail.

White matter changes also are frequent in GA-I and seem to increase with age, both in patients with high- and low-excreting biochemical phenotypes [11]. It has been recently demonstrated that manifestation of neuroaxonal impairment correlates with cerebral GA concentration and is more often found in high excretors [36]. It has already been shown for metachromatic leukodystrophy and maple syrup urine disease that white matter changes can lead to neurobehavourial and emotional disturbances, impairment of information processing, executive function and memory [28]. However, relevance of white matter changes in GA-I still remains to be elucidated, since MRI changes of white matter have also been observed in asymptomatic GA-I patients being diagnosed by newborn screening and prospectively treated [11, 49].

It is well established that GA-I patients are at risk for striatal degeneration with or without the onset of an acute encephalopathic crisis. Additionally, extrastriatal abnormalities such as pallidal hyperintensity or other deep gray matter changes frequently occur and might increase with age [11]. The relevance of both striatal and extrastriatal abnormalities to cognitive functioning is not known. Basal ganglia play an important role in neuronal circuits coordinating several brain functions including movements and behaviour [31], decision making [50], executive function, motivation and sensory and attentional activity [27] as well as rapid combination of information from multiple stimuli [51]. Dystonia is the consequence of striatal degeneration in GA-I [11, 15, 49]. Given the overall positive results in tests without time constraints and higher cognitive load in dystonic patients highlights that effects of loss of basal ganglia function, frontotemporal hypoplasia, and white matter disease on cognitive function needs further investigation.

In addition to inborn and acquired structural cerebral abnormalities cognitive function in GA-I might also be affected by disease-specific pathomechanisms: accumulating neurotoxins glutaric acid and 3-hydroxyglutaric acid might chronically affect glutamatergic signalling and thus further increase the risk for cognitive impairment [11, 27, 29–31]. Affection of cortical circuits and networks due to disturbed glutamatergic signalling might decrease processing speed but also affect memory functions due to disturbance of excitatory postsynaptical potentials (EPSP) that play an important role in memory processes.

Interestingly, in spite of several harmful structural and metabolic cerebral abnormalities in GA-I patients we have not identified clear-cut differences in non-speed facets between asymptomatic and symptomatic patients and controls. Since all patients have received metabolic treatment, this positive result speaks in favour of adherence to current guideline recommendations [52]. However, it remains to be elucidated whether intrinsic compensatory mechanisms help sustaining normal cognitive development in GA-I patients. Such mechanisms could be exploited for treatment.

### Limiting factors: study design aspects

Systematic evaluation of developmental functions of information processing, cognition, memory, and behaviour with sufficient sample sizes is a challenge in rare metabolic diseases like GA-I. Although our sample is quite large, we have to admit some limiting factors.

First, our study might underestimate the effect of severe dystonia. The sample included only three severely dystonic patients of which even one finished all subtests while the other two patients did not finish only one respectively two subtests. Furthermore, the rate of unfinished subtests was the same in asymptomatic and symptomatic patients. A larger group of severely dystonic patients will be necessary to further investigate the hypothesis that intellect is preserved in GA-I. Second, age groups had unequal sizes, sometimes with few or even no patients especially in older groups. Therefore, we cannot exclude false-negative findings. However, data of ten age groups were fitted successfully to the negative exponential model, exploiting the information of the whole data set covering a long age period. Third, new test designs should include even more complex cognitive tasks comprising intelligence tests, avoiding the motor component in output measurement, e.g. by using eye movement detection. Fourth, our findings result from a cross-sectional analysis but the silver bullet for developmental research is the longitudinal design, although hard to conduct. Last not least, but more realistic, studies combining psychological variables with (f)MRI investigations will help to understand the impact of cerebral abnormality on the neuropsychological phenotype of GA-I.

## Conclusion

In conclusion, this first evaluation of different tasks of information processing in a larger group of GA-I patients has demonstrated that dystonia predominantly impairs performance in motor speed tasks but not in tests with higher cognitive demand. Patients without motor symptoms did not differ from controls. Developmental functions of patients and controls fitted well to a negative exponential model and were similar. These findings suggest that cognitive function might be preserved in GA-I, even in patients with striatal degeneration, and favours adherence to current guideline recommendations.

## Additional file

Additional file 1: Table S1. Age groups of asymptomatic and dystonic GA-I patients and controls per subtest. (DOCX 19 kb)

## Abbreviations

3-OH-GA: 3-hydroxyglutaric acid
AICc: Akaike Information criterion bias-corrected
BADS: Barry-Albright Dystonia Scale
C5DC: Glutarylcarnitine
CSF: Cerebrospinal fluid
CP: Continuous performance
DT: Dual task
GA: Glutaric acid
GA-I: Glutaric aciduria type I
GCDH: Glutaryl-CoA dehydrogenase
GC/MS: Gas chromatography/mass spectrometry
NBS: Newborn screening
Q1: 25^th^ percentile
Q3: 75^th^ percentile
PKU: Phenylketonuria
RSS: Residual sum-of-squares
SDS: Standard deviation score
SE: Standard error of the mean
SRT: Simple reaction time
VS: Visual search
VWM: Visual working memory
