# Supplementary material for: A cross-sectional controlled developmental study of neuropsychological functions in patients with glutaric aciduria type I
Source: Orphanet J Rare Dis. 2015 Dec 22;10:163. doi: 10.1186/s13023-015-0379-6 (PMC4689061; doi:10.1186/s13023-015-0379-6)
Supplement: Additional file 1: Table S1. — Age groups of asymptomatic and dystonic GA-I patients and controls per subtest. (DOCX 19 kb) [file 13023_2015_379_MOESM1_ESM.docx]

|  |  |  |  |  |  |  |  |  |  |  |
| --- | --- | --- | --- | --- | --- | --- | --- | --- | --- | --- |
|  | **Age group [years]** | | | | | | | | | |
|  | **5-6** | **7-8** | **9-10** | **11-12** | **13-14** | **15-17** | **18-20** | **21-23** | **24-26** | **27-29** |
|  | n | n | n | n | n | n | n | n | n | n |
| **SRT** |  |  |  |  |  |  |  |  |  |  |
| Dystonic patients | 3 | 2 | 1 | 2 | 2 | 0 | 2 | 0 | 1 | 0 |
| Asymptomatic patients | 7 | 3 | 1 | 1 | 2 | 2 | 0 | 0 | 0 | 1 |
| Controls | 10 | 22 | 18 | 29 | 53 | 23 | 11 | 13 | 10 | 7 |
| **CP** |  |  |  |  |  |  |  |  |  |  |
| Dystonic patients | 3 | 2 | 1 | 1 | 1 | 0 | 2 | 0 | 1 | 0 |
| Asymptomatic patients | 6 | 3 | 1 | 1 | 2 | 2 | 0 | 0 | 0 | 1 |
| Controls | 9 | 21 | 9 | 27 | 53 | 23 | 11 | 13 | 10 | 7 |
| **Tracking** |  |  |  |  |  |  |  |  |  |  |
| Dystonic patients | 3 | 2 | 1 | 2 | 2 | 0 | 2 | 0 | 1 | 0 |
| Asymptomatic patients | 7 | 3 | 1 | 1 | 2 | 2 | 0 | 0 | 0 | 1 |
| Controls | 10 | 15 | 12 | 15 | 15 | 18 | 7 | 11 | 9 | 7 |
| **VS3** |  |  |  |  |  |  |  |  |  |  |
| Dystonic patients | 0 | 1 | 1 | 2 | 1 | 0 | 2 | 0 | 1 | 0 |
| Asymptomatic patients | 3 | 3 | 1 | 1 | 2 | 2 | 0 | 0 | 0 | 1 |
| Controls | 3 | 18 | 9 | 26 | 53 | 23 | 11 | 12 | 10 | 7 |
| **VWM** |  |  |  |  |  |  |  |  |  |  |
| Dystonic patients | 1 | 2 | 1 | 2 | 2 | 0 | 2 | 0 | 1 | 0 |
| Asymptomatic patients | 6 | 3 | 1 | 1 | 2 | 2 | 0 | 0 | 0 | 1 |
| Controls | 9 | 15 | 18 | 17 | 15 | 18 | 7 | 10 | 9 | 7 |
|  |  |  |  |  |  |  |  |  |  |  |

**Supplementary Table 1.** Age groups of asymptomatic and dystonic GA-I patients and controls per subtest.
